# The impact of information accuracy on the selective trust of children aged 3–6

**DOI:** 10.3389/fpsyg.2025.1539242

**Published:** 2025-07-07

**Authors:** Shu Mou, Xiu Mu, Nan Wu, Yifang Wang

**Affiliations:** ^1^Department of Psychology, Teachers’ College of Beijing Union University, Beijing, China; ^2^Psychology Department, City University of Macau, Macao, Macao SAR, China; ^3^College of Preschool Education, Capital Normal University, Beijing, China

**Keywords:** selective trust of children, information accuracy, interpersonal safety skills, anti-abduction knowledge, 3–6 year olds

## Abstract

Promoting young children’s selective trust ability and interpersonal safety knowledge is the goal of many child educational administrations and maltreatment prevention programs, but questions remain about the factors that influence the development and effectiveness of these skills. The goal of this study was to explore the impact of information accuracy on selective trust in children aged 3–6 years as well as the roles of informant gender and safety knowledge. Using a real-world contextual experimental paradigm, we conducted two experiments using a 2 (age group: 3–4 years vs. 5–6 years) × 2 (information accuracy: accurate vs. inaccurate) between-subjects design to examine whether children would follow a female/male stranger when confronted with accurate/inaccurate information and to measure the length of time the children hesitated when making the decision to leave. The role of safety knowledge in the decision process was also tested by questionnaire. We found that: (1) information accuracy significantly influenced selective trust among 5–6-year-olds; (2) regardless of whether the information was accurate or not, children aged 5–6 years hesitated longer than children aged 3–4 years in choosing to leave; (3) more 3–6-year-olds chose to trust a female stranger than a male stranger; and (4) 5–6 year-old children’s safety knowledge about prevention of abduction has a protective role in selective trust.

## Introduction

In the early years of life, children begin to learn many skills and facts from other people. Central challenges that children face in learning from others are deciding from whom to learn and determining the reliability of the source. The ability to confirm or question the reliability of others and their information is known as selective trust (Harris et al., 2012). Selective trust not only guarantees the establishment and maintenance of healthy interpersonal relationships, but it also promotes interpersonal safety knowledge that can help children avoid unsafe stranger interactions and child trafficking (White et al., 2018).

The rapid changes in technology that have enhanced social connections and information leakage are unavoidable, and they have been accompanied by unsafe stranger interactions and child trafficking throughout the world. This crime is a lucrative trade that causes mental and physical harm to victims and their families, and it is also a serious social problem that jeopardizes the stability of society. According to the National Crime Records Bureau 2021 in India, 2,877 children were trafficked for manual and sexual labor, 77,535 children were reported missing (ncrb.gov.in), and 37% of those children were trafficked by means of fraud and deception (e.g., expressing familiarity, such as knowing their name, address, and relatives) (United Nations Inter-Agency Project [UNIAP], 2015). These data highlight the importance of developing children’s selective trust ability and providing relevant educational interventions for families and schools to enhance children’s ability to discern safe *vs*. unsafe situations (Kim and Harris, 2014; Dimitropoulos et al., 2022). However, questions remain about the factors that influence children’s trust and the effectiveness of interpersonal safety knowledge and skills.

The development of selective trust emerges in infancy. Initially, children exhibit naïve trust rather than sophisticated reasoning, as indicated by results of many one-informant studies in which children were presented with one speaker who consistently labeled familiar objects either accurately or inaccurately. Such studies showed that children tend to generally trust others, even those who have proven consistently unreliable in the past. For example, 2-year-olds kept following the hints of an unreliable informant even when they conflicted with their own experience (Jaswal et al., 2014), and 3-year-olds continued to endorse cues, even after eight trials of misinformation about the location of a hidden object (Jaswal et al., 2010). However, when the conflicting sources paradigm was used, even 18-month-olds showed a preference for reliable information providers and accurate sources of information (Luchkina et al., 2018). Other studies reported that children as young as age 3 displayed greater trust in one informant over others, such as a person who accurately labeled familiar objects in the past (Koenig et al., 2004; Koenig and Harris, 2005) or who showed confidence when speaking (Bridgers et al., 2016).

Despite the different paradigms used, most studies make it clear that by 4 years of age, if not younger, children show a robust preference for learning from reliable sources, and this trait becomes stable at age 5 (Poulin-Dubois and Brosseau-Liard, 2016; Sampaio et al., 2019). This is relevant to the development of children’s cognitive abilities and also marks an accomplishment in their interpersonal skills (Harris et al., 2012). Other complex variables also influence children’s selective trust competence, such as their enrollment in kindergarten.

Factors affecting children’s selective trust can be either external or internal. Safety awareness in an internal factor (Li et al., 2014). Research indicates that by around age 3, children begin to develop selective trust with the increase of skills and knowledge. In particular, their increasing knowledge about safety and interpersonal skills enhances their competence to discern and evaluate information, thereby improving their selective trust and personal defense in unfamiliar and unsafe situations (White et al., 2018). External factors are more related to the information provider (Nadarevic et al., 2020). They include the accuracy of the information (Luchkina et al., 2020) and the identity of the informant (Corriveau et al., 2009; Ma et al., 2017) and may be related to gender or social status.

The accuracy of the information given by various informants can influence children’s selective trust (Mirtaheri et al., 2022). Previous studies suggest that by ages 5–6, children tend to trust those who provide accurate information regardless of the intent of the informant (Lane et al., 2013; Tong et al., 2020). Koenig et al. (2004) employed a classic item-naming task paradigm to examine the effects of information accuracy on children’s trust, in which accuracy was defined as entirely accurate *vs.* completely wrong. Results showed that 5–6-year-olds trusted informants who were entirely accurate. Other findings showed that 3–4-year-olds would still follow a stranger irrespective of the accuracy of the information and that 5–6-year-olds followed strangers more frequently only when given accurate information (Li et al., 2020). Furthermore, Li et al. (2020) found that 5–6-year-olds were significantly more likely to leave with her when her information was accurate rather than inaccurate, and those who chose to leave were significantly more reluctant to do so in the inaccurate condition. One possible explanation for this finding is that children may consider inaccurate informants to be untrustworthy with reference to their knowledge of anti-trafficking education experience, but this premise needs to be further clarified in a real kindergarten setting. Numerous cases of child abductions indirectly support these experimental results, as criminal suspects often deceive younger children using accurate information, such as correctly stating the child’s name, age, or parents’ occupations. Therefore, the main goal of many child maltreatment programs is to promote young children’s interpersonal safety knowledge, intentions, confidence, and information identification skills (White et al., 2018).

The identity of the information provider is also a significant variable that influences young children’s selective trust, and the role of authority and gender of the informant on children’s behavior has been studied previously. Young children are more likely to perceive information from authoritative figures as reliable and are more inclined to trust them. In one study, compared to older peers, 3–5-year-olds preferred to trust answers given by teachers and displayed consistent trust across various situations (Ma et al., 2017).

Li et al. (2020) investigated the impact of the informant’s gender on children’s selective trust. After establishing personal trustworthiness with the participants by providing them with information they knew to be correct, the experimenter invited the children to leave with him/her. The results revealed that when the experimenter was female, 3–6-year-old children were more inclined to follow. This finding is consistent with data from many legal cases related to child abduction, which show that women are often active players in trafficking operations. For example, in a study conducted in India, Nair (2004) found that nearly half of the trafficked persons reported that their traffickers were women. A United Nations study reported that about 28% of convicted traffickers were women (United Nations Office on Drugs and Crime [UNODC], 2014), whose role in the children-trafficking process is frequent interaction with victims. Shen and Winlow (2013) suggested that the majority of child-trafficking incidents in China involve women as perpetrators who play a recognizable role throughout the trafficking processes.

In summary, information accuracy and the identity of the information provider affect selective trust across different age stages, but how they function in real-world scenarios needs requires further exploration. Furthermore, interpersonal safety skills and knowledge actually do increase children’s rate or timing of disclosure of risky situations (White et al., 2018). We hypothesize that children’s knowledge about trafficking might increase their selective trust. Therefore, we conducted two experiments in a real kindergarten setting to examine the effects of information accuracy and identity of the information provider on selective trust in 3–6-year-olds. In both experiments, children’s knowledge about abduction prevention was also assessed to examine its impact on children’s trust and its protective role in staying safe.

## Experiment 1

### Materials and methods

#### Participants

Study participants were recruited from a kindergarten partnering with the research team on an anti-trafficking safety education program. A chi-square (Goodness-of-fit tests: Contingency tables) power analysis in G* Power 3 (Faul et al., 2007) indicated a minimum total sample size of 58 to achieve appropriate power to detect the effect size as Li et al. (2020) (parameters: effect size ω = 0.37, alpha = 0.05, power = 0.8 Df = (2−1)*(2−1) = 1). We recruited a total of 60 participants, with 30 in the 3−4 year-old age group (*M* = 3.83, *SD* = 0.32) and 30 in 5−6 years old (*M* = *5.85, SD* = 0.26). Before the experiment, we obtained informed consent from the parents and investigated whether the children’s parents and kindergarten had provided education related to anti-trafficking.

The present experiment was approved by the Ethics Committee of the Psychology Department at Beijing Union University, and a written consent was obtained from each child’s parents prior to the experiment.

#### Experimental design and materials

This experiment was adapted from the real-life experimental paradigm by Li et al. (2020), using a 2 (age groups: 3−4, 5−6 years) × 2 (information accuracy: accurate, inaccurate) between-subjects experimental design. The independent variables are information accuracy and age group, while the dependent variable was the trust-related decisions (whether the child leaves and the hesitation time). Children were randomly assigned to either an accurate information group or an inaccurate information group.

The experiment was conducted in a quiet classroom of the kindergarten. One wide-angle camera and one close-up camera were used to film the entire experimental process to accurately record the children’s performance (whether they left, hesitated, hesitation time etc.).

#### Experimental tasks and procedures

At the start of the experiment, a teacher led the child to the experimental room, where a female assistant played with the child (using a ball or doll). During that time, the assistant’s phone rang. She told the child she was going to go outside to answer it and asked the child to play alone for a while, then she left the room. After a minute, a female stranger (another female experimenter) entered, initiated playing with the child, and proceeded with the following:

Accurate Information Group:

(1) The stranger provided accurate information about the child’s parents’ names and extended an invitation: “I know your mom, her name is xxx (correct name),” and “I also know your dad, and his name is xxx (correct name).” “You are so cute, I really like you, I have a gift for you, let’s go to the car outside the kindergarten to get it, I’ll bring you back soon.” The female experimenter then stood up and invited the child to go with her.
(2) Upon extending the invitation to leave, the experimenter started the stopwatch, recording the time from when the invitation was made to when the child agreed or refused to leave the classroom.
(3) Afterward, the child answered the following questions:
  (a) Did your parents teach you not to leave with strangers?
  (b) Did your kindergarten teacher teach you not to leave with strangers?

Inaccurate Information Group:

(1) The stranger provided incorrect information about the child’s parents’ names and extended the same invitation to leave. If the child questioned the information, the experimenter explained: “Ah, maybe I got it wrong, but I do have a gift for you, let’s go to the car outside the kindergarten.”
(2) and (3) followed the same procedure as in the accurate information group.

At the end of the experiment, the female experimenter provided emotional support and gave each child an eraser as a gift. The following day, the experimental scenario was incorporated into a comprehensive safety education session, where it served as a practical example to enhance children’s understanding of interpersonal safety principles and to facilitate the generalization of protective behaviors across various potentially unsafe social situations.

#### Coding

The child’s behavior was recorded as follows: (a) whether they left classroom, and (b) for those who left, the hesitation time before leaving (a longer hesitation time indicated that the child spent a longer time considering whether or not they should trust the stranger). Regarding knowledge about avoiding abduction, if the child answered “yes” to both questions in (3) and these questions were marked “yes” in the survey of their parents and teachers, they were coded as having knowledge about anti-abduction. Otherwise, they were coded as having “no” knowledge about anti-abduction.

### Results for experiment 1

#### Effects of information accuracy and age on selective trust with a female informant

In this experiment, 68.8% of 3–4-year-old children left with the informant under accurate information conditions, and 57.1% of them left under inaccurate conditions. For the 5–6-year-old age group, 46.7% of the children left with the informant under accurate conditions, but only 1 child left under inaccurate conditions.

**FIGURE 1:**
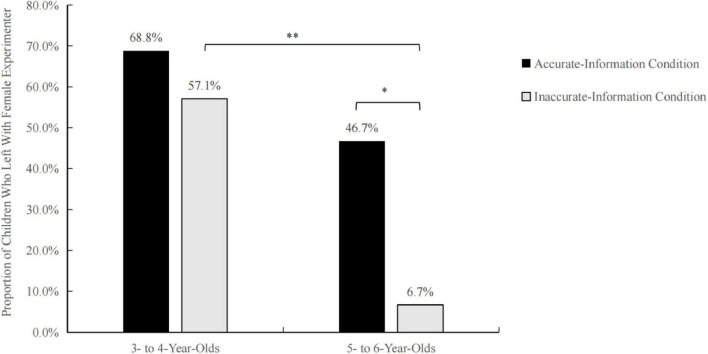
Proportion of children in each age group and information accuracy condition who left with the female experimenter. *P < 0.05, **P < 0.01.

We used a chi-square test to compare the influence of information accuracy on the selective trust of the two groups of children (Figure 1). The number of 3–4-year-old children who left with the informant did not differ significantly between the information accuracy conditions (*p* = 0.510). However, for the 5–6-year-old children, the number of departures differed significantly between the information accuracy groups [*χ^2^*(1, 30) = 6.136, *p* = 0.013]. Children in the group with accurate information chose to leave significantly more than those in the group with inaccurate information. In this experiment, in which the informant was female, the accuracy of the information had little impact on the 3–4 age group children. For 5–6 year-olds, however, the more accurate the information, the more likely they were to trust and leave with the informant. When given accurate information, the number of departures did not differ significantly between the two age groups (*p* = 0.213), whereas significantly more 3–4 year-olds than 5–6 year-olds left with the informant when the information was inaccurate [*χ^2^*(1, 29) = 8.620, *p* = 0.003]. This result shows that more 5–6 year-olds refused to leave with a stranger when faced with inaccurate information.

**FIGURE 2:**
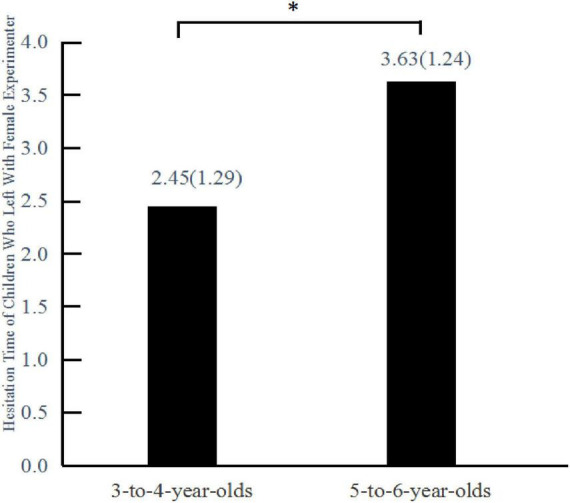
Mean hesitation time of children who chose to leave with the female experimenter in Experiment 1. Hesitation time is given in seconds; values in parentheses after the mean values are standard deviations. *P < 0.05.

The Mann-Whitney U test used to assess the hesitation time of those children who chose to leave with the female experimenter revealed a statistically significant difference in hesitation time between the two age groups (*U* = 33.0, *Z* = –2.28, *p* = 0.022) (Figure 2). The hesitation time of 5–6 year-olds (*M* = 3.63, *SD* = 1.24) was significantly longer than that of 3–4 year-olds (*M* = 2.45, *SD* = 1.29).

#### Effects of relevant anti-abduction knowledge on selective trust

Analysis of the relationship between anti-abduction knowledge and children’s selective trust revealed that 32 of the children who refused to leave (97.0%) had such knowledge. Among those who chose to leave, 10 children (46.2%) had anti-abduction knowledge, and 14 children (53.8%) did not. The chi-squared showed children who accumulated anti-abduction knowledge are more likely to refuse to leave, *χ^2^* (1, *n* = 59) = 19.806, *p* < 0.001.

## Summary of experiment 1

The results of Experiment 1 showed that the more accurate the information provided, the more children in both age groups trusted the female stranger and the more likely they were to leave with her (30%). This is consistent with the findings of previous research on selective trust (Pasquini et al., 2007; Guerrero et al., 2020). Among the 5–6 year-olds, more children left when the information was accurate. Additionally, of those who chose to leave with the informant, 5–6 year-olds hesitated significantly longer than did the 3–4 year-olds. This suggests that 5–6 year-olds have significantly better information discernment abilities than 3–4 year-olds. With inaccurate information, more 5–6 year-olds refused to leave compared to 3–4 year-olds, and children who had anti-abduction knowledge were more likely to refuse to leave.

Our results indicate that from the perspective of children’s cognitive abilities and development of information judgment, 5–6 year-olds who are aware of abduction risk can better combine on-site information and previous knowledge to form comprehensive clues for rational trust judgments. These results are consistent with those of previous studies (White et al., 2018; Rakoczy et al., 2022). To verify whether the above conclusions apply to male strangers, Experiment 2 was conducted.

## Experiment 2

### Methods

#### Participants

Similar as experiment 1, a total of 60 children aged 3−6 were recruited from a kindergarten (30 boys). Among them, there were 30 children (15 boys) in the age group of 3−4 year-olds (*M* = 3.75, *SD* = 0.39) and 30 children (15 boys) aged 5−6 years old (*M* = 5.96, *SD* = 0.42). Before the experiment, we obtained informed consent from the parents and investigated whether the children’s parents and kindergarten had provided education related to anti-trafficking.

#### Experimental design and materials

The same as Experiment 1.

#### Experimental task, procedure, and coding

The experimenter changed to a male. The rest of the procedure and coding was the same as Experiment 1.

### Results of experiment 2

#### Effects of information accuracy and age on selective trust with a male informant

Descriptive statistics were used to compare the number and percentage of children in each age group who left with the male informant under accurate and inaccurate information conditions. Among the 3–4-year-olds, 7 (46.7%) left with the experimenter under the accurate information condition and 11 (73.3%) left under the inaccurate information condition. For the 5–6 age group, 4 (26.6%) and 1 (6.7%) children left with the experimenter under the accurate and inaccurate information conditions, respectively.

**FIGURE 3:**
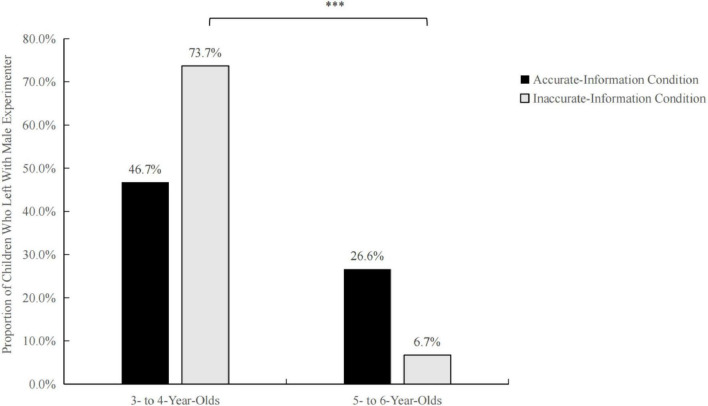
Proportion of children in each age group and information accuracy condition who left with the male experimenter. ***P < 0.001.

The chi-square analysis of the effects of information accuracy on children of different ages showed that there was no significant difference in the number of departures between the two age groups under the accurate information condition (*p* = 0.2) (Figure 3). When inaccurate information was provided, 3–4-year-olds were significantly more likely to leave and 5–6-year-olds were more likely to refuse to leave (*p* = 0.001).

**FIGURE 4:**
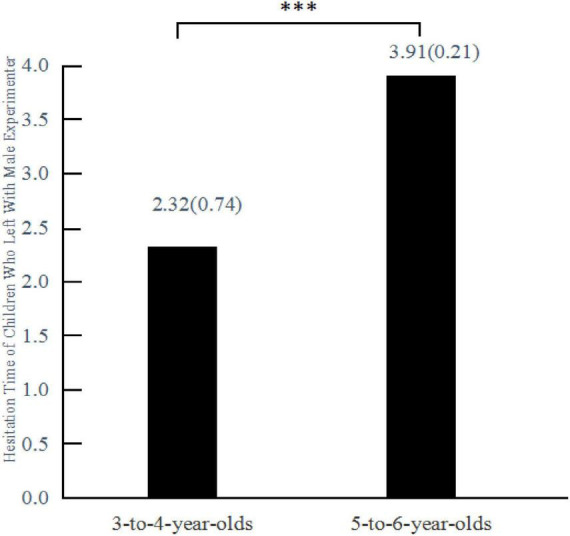
Mean hesitation time of children in each age group who chose to leave with the male experimenter in Experiment 2. Hesitation time is given in seconds; values in parentheses after the mean values are standard deviations. ***P < 0.001.

The Mann-Whitney U test used to assess the hesitation time of those children who chose to leave with the male experimenter revealed a statistically significant difference in hesitation times between groups [*U* = 1.50, *Z* = –3.24, *p* < 0.001] (Figure 4). The hesitation time of 5–6-year-olds (*M* = 3.91, *SD* = 0.21) was significantly longer than that of 3–4-year-olds (*M* = 2.32, *SD* = 0.74).

#### Effects of relevant anti-abduction knowledge on selective trust

The analysis of the relationship between children’s knowledge of abduction risk and selective trust revealed that all 34 children who refused to leave had anti-abduction knowledge. Among those who chose to leave, only 6 (26.1%) had anti-abduction knowledge, while 17 (73.9%) did not. Further chi-square analysis showed a significant difference [*χ^2^*(1, 57) = 35.811, *p* < 0.001], indicating that when faced with a male stranger, anti-abduction knowledge significantly enhanced children’s information recognition and judgment capabilities.

## Summary of experiment 2

**FIGURE 5:**
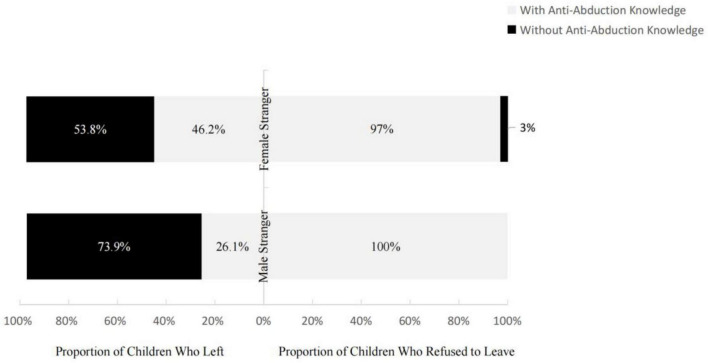
Proportion of children who left with strangers of different genders (left side) and those who refused to leave (right side).

The results of Experiment 2 showed that when the stranger was male, more 5–6-year-olds refused to leave with him than did 3–4-year-olds when the information was inaccurate. This further validates the results of Experiment 1 and is consistent with previous research results on selective trust in male strangers (Li et al., 2020). Among those who chose to leave with the male informant, 5–6-year-olds hesitated for a longer time, especially when the information was accurate, which was consistent with the results of Experiment 1 with a female stranger. In addition, compared to the role of anti-abduction knowledge in the face of the female stranger in Experiment 1, children who knew about abduction risk all refused to leave when facing a male stranger (Figure 5). This is consistent with existing research results (Finkelhor et al., 2021).

## Discussion

In this study, we explored the influence of information accuracy, the information provider’s gender, and anti-trafficking knowledge on the selective trust of children aged 3–6 through real-world experiments. The results showed that regardless of whether the stranger was female or male, the accuracy of the information significantly affected the selective trust of these children. The effect of information accuracy was greater in the older group (5–6-year-olds), and female strangers were viewed as more trustworthy than male strangers in unsafe situations. Moreover, anti-trafficking education enhanced children’s selective trust capabilities, which indicates that such knowledge can help keep them away from unsafe situations to some extent.

Our finding that information accuracy has a significant impact on children’s selective trust is consistent with previously reported data (Butler et al., 2020; Li et al., 2020). Specifically, when the information was inaccurate, we found that the majority of 5–6-year-olds refused to leave with the stranger, regardless of the stranger’s gender. Among those who chose to leave, 5–6-year-olds hesitated longer than 3–4-year-olds, especially when the information was inaccurate.

We propose that this field study illustrated the sustained effect of information accuracy on selective trust for at least two reasons. First, a crucial cognitive development shift occurs in children around the age of 4–5, especially in terms of their theory of mind and executive function development, which helps children make more effective choices in trust scenarios (Miyoshi and Sanefuji, 2020). Previous research has shown that the better the development of a theory of mind, the stronger the ability to infer the mental states of others and, consequently, the better the ability to infer others’ behaviors and underlying mental states. The age of 4–5 is a period of rapid development of theory of mind, during which children begin to more accurately interpret the verbal information in lies and infer whether the actions and intentions of others are benevolent or malevolent, which in turn allow them to make more rational trust decisions (Michael and Breaux, 2021; Souza et al., 2021). The higher level of selective trust and resistance to abduction demonstrated by 5–6-year-olds compared to 3–4-year-olds in this study supports this notion.

In the study context, children also need to exert inhibitory control to resist the temptation (gifts) of strangers (i.e., to resist dominant responses and refuse to follow strangers). The ages of 5–7 are critical periods for the development of children’s inhibitory control abilities (Munakata et al., 2012). This idea was validated by the results of Experiments 1 and 2, in which over 60 and 80%, respectively, of 5–6-year-olds refused to leave with the stranger. Compared to the younger children, those 5–6-year-olds who chose to leave spent more time evaluating the intentions of the stranger and resisting the enticing rewards offered by the stranger.

The second possible explanation for the sustained effect of information accuracy on selective trust is that children’s information judgment abilities contribute to their selective trust. As children grow older, their metacognitive abilities continue to strengthen, making them sensitive to subtle differences in cues when analyzing information providers and sources. They can reflect upon and revise their beliefs and perspectives based on social cues (Resendes et al., 2021). Trust judgments, which are more influenced by social factors in early years, gradually become more influenced by individual cognitive abilities and knowledge-based factors (such as information accuracy) (Li et al., 2020). Four-year-old children can make reasonable trust judgments based on information accuracy and are more likely to trust information providers with higher accuracy (Heyman, 2008; Guerrero et al., 2020). This ability to judge wrong and misleading advice stabilizes at the age of 5 (Heyman et al., 2013). In our study, regardless of the gender of the stranger, more 5–6-year-old children chose to leave with the stranger when the family information was accurate *vs*. inaccurate, which seems to support this reasoning.

The gender of the informant also influences children’s selective trust. Li et al. (2020) reported that children viewed female informants as more reliable and honest than males, and results of the current study showed that regardless of the accuracy of the information, children aged 3–6 were more likely to refuse to leave with male than with female experimenters. A possible explanation for this result might be that infants reared with a female primary caregiver prefer female over male faces and gradually become more wary of males (Quinn et al., 2002; Sugden and Moulson, 2019). Conceptually, from the perspective of evolutionary theory, humans evolved to perceive men as more aggressive and women as more trustworthy because of reproductive fitness and mate selection, and societal and cultural factors have reinforced this pattern (Stirrat and Perrett, 2010). We found that 23.3% of 5–6-year-old children chose to trust and follow the female experimenter who provided accurate information, which in some ways supports this explanation. Our results are consistent with real-life legal cases, which show that children are at greater risk of deception and abduction when confronted with verbal and material lures from unfamiliar females compared to male strangers (Shen and Winlow, 2013).

In contrast to findings reported by Li et al. (2020), the 3–4-year-olds in our study were actually more likely to trust an inaccurate male informant (73%) compared to an inaccurate female informant (57%). Scarcity theory provides one possible explanation for this finding, as there are significantly fewer male teachers than female teachers in the field of early childhood education. This situation of “having” less than they “need” may capture young children’s attention and create a “scarcity mentality” that may develop into an attentional bias toward the more novel speaker. If children consider them and what they say to be funny and interesting, they may more readily opt to leave with the male informant (Mullainathan and Shafir, 2013; Heyes, 2017). Furthermore, at the time when we conducted our study, two male teachers had just begun to work full-time in the kindergarten. The participants’ emotional attachment to and bonding with them might have positively affected their trust in the male experimental assistant in our study. Another explanation is that younger children’s judgment reflect superficial impression rather than sophisticated reasoning, they often selectively learn from a strong and an attractive model rather than a weak and an unattractive one (Jaswal and Kondrad, 2016; Hermes et al., 2018). In our study, the male informant is taller and stronger than the female informant, these superficial cues might be endorsed by younger children.

White et al. (2018) reported that anti-trafficking education enhances children’s safety discernment, and we also found a connection between children’s knowledge about abduction risk and children’s selective trust. Regardless of the accuracy of the information and the gender of the experimenters, most children aged 3–6 who refused to leave had anti-abduction knowledge. Even among children who failed to offer a verbal refusal, those with anti- abduction knowledge hesitated for a longer time than those without it. Previous studies have indicated that children’s resistance to abduction is influenced not only by their cognitive development but also by daily anti-abduction education from parents and teachers. Frequent reminders of safety knowledge and skills from parents and teachers are a common method in daily interpersonal safety and privacy education (Vanderbilt et al., 2014). Such reminders enhance children’s memory of related events, and the warnings strengthen their attitude toward them (Beuscher and Roebers, 2005). Hence, in an era in which personal privacy breaches are inevitable due to technology advancements, regularly imparting safety knowledge and skills to children is crucial (Roscoe et al., 2021), especially for young preschool children with immature cognitive abilities and limited social experiences. Proper anti-abduction education could be a protective factor that would assist children in making better trust judgments (Finkelhor et al., 2021).

In summary, our two field experiments showed that nearly half of the children aged 3–4 chose to leave with a stranger, while a smaller proportion of children aged 5–6 made the same choice. This result shows that preschool children, especially those aged 3–4, are easily deceived, which is consistent with early research showing that children aged 3–4 years have difficulty refusing abduction lures, with 90% being persuaded to leave in simulated stranger studies (Goldfarb et al., 2008; White et al., 2018). Hermes et al. (2018) theorized that young children tend to generally trust others, even those who have proven consistently unreliable in the past. The way young children learn from others often appears to reflect naïve trust (a fast, implicit, and heuristic cognitive process) rather than sophisticated reasoning. Our results also showed that children aged 3–6 trust female strangers more than male strangers and that anti-abduction knowledge is a protective factor that influences children’s selective trust.

This study also had some limitations. First, in addition to the experimenter’s gender and the accuracy of the information provided, other factors such as the experimenter’s familiarity, facial attractiveness, and the background of the information provided affect children’s selective trust and require further investigation. Second, although the study imitated real-life abduction situations, it was still conducted within a kindergarten, and the children may have viewed the informants as staff members. Their emotional bonding with the teachers might have positively affected the reliability of the evaluation of the female and male informants, which could explain why 57 and 75% of 3–4 years old children trusted inaccurate information provided by the female and male informants, respectively. The improved trust when encountering the inaccurate speaker may have been moderated by the familiarity or predictability of the kindergarten situation rather than by epistemic trust of a specific person. Therefore, it is unclear whether the children’s connection to the kindergarten may have reduced their vigilance and the validity of our findings. Experiments conducted in parks or malls might overcome this limitation and improve the ecological validity. Lastly, directly refusing others is seen as rude and impolite in Chinese traditional etiquette, and this could have influenced children’s responses to the abduction lure in our study (nearly half of the 3–4-year-old children left with the experimenter). A cross-culture experimental design may help clarify these confounding variables.

## Conclusion

We designed two experiments to explore the role of information accuracy, gender of informants, and anti-abduction knowledge in the development of children’s selective trust. We found that the more accurate the information, the more likely the children were to trust strangers and that the children were more likely to selectively trust female strangers compared to male strangers. Furthermore, compared to 3–4-year-old children, 5–6-year-olds demonstrated a marked improvement in information discernment and risk identification in the ambiguous trust situation. Notably, anti-trafficking education enhanced the children’s discernment of safety/risk.

## Data Availability

The original contributions presented in this study are included in this article/supplementary material, further inquiries can be directed to the corresponding author.
